# Range Sizes of the World’s Mammals, Birds, and Amphibians from the Mid-Holocene to the Industrial Period

**DOI:** 10.3390/ani11123561

**Published:** 2021-12-15

**Authors:** Robert Beyer, Andrea Manica

**Affiliations:** 1 Department of Zoology, University of Cambridge, Downing Street, Cambridge CB2 3EJ, UK; am315@cam.ac.uk; 2 Potsdam Institute for Climate Impact Research (PIK), Member of the Leibniz Association, Telegrafenberg A 31, 14473 Potsdam, Germany

**Keywords:** biodiversity, climate change, land use, range shifts, palaeoclimate, agriculture

## Abstract

**Simple Summary:**

The geographic ranges of animal species play an important role for many ecological processes. Changes in climate, as well as human conversion of natural habitats, are two major factors that affect species’ range sizes. While the impact of these two factors in the Industrial era has been thoroughly studied, their pre-Industrial impacts are less well understood further back in time. Here, we combine global reconstructions of land use and climate from 6000 BCE to 1850 CE with data of the geographic distributions and habitat requirements of 16,919 mammal, bird, and amphibian species to estimate how human land use and natural climatic change have altered species’ ranges across past millennia. Our results suggest that pre-Industrial land use had only a small impact, yet one that affected almost all species negatively. Climatic variation evidently led to some range expansions and contractions, but overall had a small impact on the majority of species. In the context of a previous study of range changes in the more recent past, our results demonstrate that current rates of range losses exceed the magnitude of range changes seen over many thousands of years prior to the Industrial period to an alarming extent.

**Abstract:**

Anthropogenic land use and climate change in the Industrial age have had substantial impacts on the geographic ranges of the world’s terrestrial animal species. How do these impacts compare against those in the millennia preceding the Industrial era? Here, we combine reconstructions of global climate and land use from 6000 BCE to 1850 CE with empirical data on the spatial distributions and habitat requirements of 16,919 mammal, bird, and amphibian species to estimate changes in their range sizes through time. We find that land use had only a small, yet almost entirely negative impact during most of the study period, whilst natural climatic variability led to some range expansions and contractions; but, overall it had a small impact on the majority of species. Our results provide a baseline for comparison with studies of range changes during the Industrial period, demonstrating that contemporary rates of range loss exceed the magnitude of range changes seen over many thousands of years prior to the Industrial period by an alarming extent.

## 1. Introduction

The geographical ranges of animal species play an important role for a number of important ecological processes and variables, such as local biodiversity and trophic dynamics [1], species’ vulnerability to extinction [2,3], and evolution [4]. Climatic change and anthropogenic land use are two major factors affecting the spatial extent of species’ habitats [5,6]. In recent times, they have both led to severe range contractions for some species [5,7,8,9,10,11] and range expansions for others [5,12], resulting, respectively, in the introduction of species into new areas and the local extinction of others. Future projections of global land use and climate change suggest that species’ geographic ranges will continue to be subject to significant shifts [5,13,14].

Whilst species range dynamics on time scales of recent decades up to centuries have been examined by a number of recent studies [5,7,8,9,10,11], climate variability and land-use-driven range dynamics in the deeper, pre-Industrial past have received less attention. However, reconstructing changes in the range sizes of the world’s species across very long time scales can provide important insights, for example into how the impact of recent and projected future anthropogenic climate change compares to that of natural climatic variability during the pre-Industrial Holocene.

Here, we combined reconstructions of global land use and climate for the last ~8000 years with empirical data on the spatial distributions and habitat requirements of 16,919 mammal, bird, and amphibian species to estimate changes in their geographic range sizes during the pre-Industrial period. We isolated the impacts of climate and land use on species’ ranges, consider range changes globally as well as for specific biomes and geographic regions, and discussed our results in the context of range size dynamics during the Industrial era.

## 2. Materials and Methods

We estimated species-specific geographic ranges through time subject to global anthropogenic land use and climatic conditions using the methodology in [5,13], summarised in Supplementary Figure S1. This model-based approach consisted of first determining the global distribution of natural vegetation corresponding to a given climate, and combining these data, as well as global reconstructions of anthropogenic land cover, with empirical datasets of the spatial distribution and habitat requirements of individual species. While land use reconstructions have been generated for as far back as 10,000 BCE, we used 6000 BCE as the starting point of our study, given that prior to this point in time, low global sea level resulted in the emergence of significant new land areas [15], occupation of which the species distribution model used here would not be able to account for.

**Figure 1:**
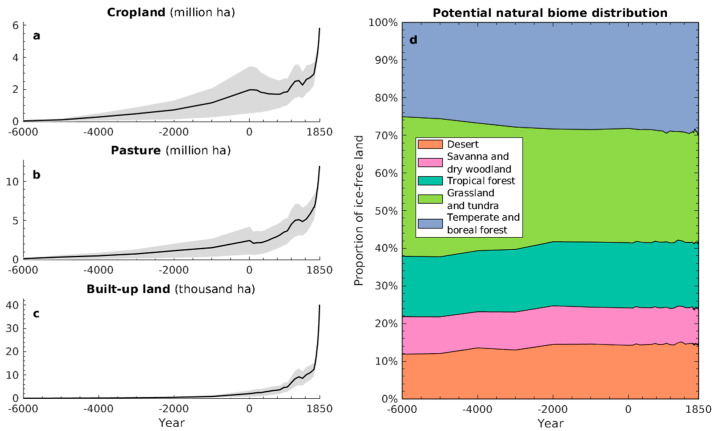
Global anthropogenic land use and potential natural vegetation between 6000 BCE and 1850 CE. (**a**–**c**) Total area of cropland, pasture, and built-up land. Grey bands represent uncertainty bounds. (**d**) Distribution of megabiomes (aggregated from the original 29 biomes simulated by Biome4, for visual clarity).

Reconstructions of global croplands, pastures, and built-up areas between 6000 BCE and 1850 CE were obtained from the HYDE 3.2 dataset [16]. A total of 43 maps were available at 1000-year time steps between 6000 BCE and 0 CE, at 100-year time steps between 0 and 1700 CE, and at 10-year time steps between 1700 and 1850 CE. These maps were upscaled from their original spatial resolution of 0.083° to a 0.5° grid. The dataset also included lower and upper uncertainty bounds for each grid cell and point in time. Figure 1a–c show the global extent of cropland, pasture, and built-up land during the study period.

To reconstruct climate-specific natural vegetation through time, we first generated global maps of monthly temperature, precipitation, and cloud cover between 6000 BCE and 1850 CE based on 3.75° × 2.5° resolution simulations of the HadCM3 climate model [17], available as climatological normals (i.e., 30-year averages) in 1000-year intervals between 6000 BCE and 1000 CE (available from: https://www.paleo.bristol.ac.uk/, last accessed on 9 December 2021), and annually for the last 1000 years (available from: https://esgf-node.llnl.gov/search/cmip5/, last accessed on 9 December 2021). Based on the latter, annual data, we generated climatological normals in 50-year intervals for the last 1000 years. The complete time series of monthly temperature, precipitation, and cloud cover normals since 6000 BCE was then bias-corrected and downscaled to a 0.5° resolution using the delta method [18] and observation-based climate maps of the 1900–1930 period [19]. Finally, the monthly climatological normals, along with reconstructions of global mean atmospheric CO_2_ concentration [20,21], were used as inputs for the Biome4 vegetation model [22], allowing us to reconstruct global biome distributions in 1000-year intervals between 6000 BCE and 0 CE, 100-year intervals between 0 and 1700 CE, and 30-year intervals between 1700 and 1850 CE. The available data did not allow us to estimate uncertainty bounds of the climate data, and by extension, the vegetation maps. Figure 1d summarises the simulated global distribution of megabiomes through time.

Following the methodology in [5,13], the geographic habitat range of each mammal, bird, and amphibian species at a given time was estimated by combining the relevant global maps of land use and natural vegetation with species-specific extents of occurrence and species-specific habitat requirements [23,24]. Extents of occurrence represent the outermost geographic limits of a species’ observed or projected occurrence [2]; these spatial envelopes do not account for the distribution of vegetation within that area and therefore generally extend substantially beyond a species’ actual distribution [25]. Habitat requirements include one or more vegetation categories in which a species can occur. We rasterised extents of occurrence from their original spatial polygon format to a 0.5° grid, and subsequently refined them by retaining only those grid cells where the previously estimated natural vegetation type, at the relevant time, was included in the species’ list of habitat requirements. The result of this step represents a species’ potential natural range at the given time, i.e., in the hypothetical absence of anthropogenic land use. Species-specific habitat requirements also contain information on the types of anthropogenic land cover, if any, that a species can live in. By combining this information with the available reconstructions of global land use through time, we estimated each species’ actual habitat range at a given time by subtracting from the previously derived potential natural range the fraction of unsuitable anthropogenic land cover in each relevant grid cell.

In addition to considering the effects of land use and climate change in combination, we also examined two scenarios in which we isolated each of these two factors. In the first scenario, we considered the impact of land use on species’ ranges through time under the global vegetation estimated for the year 1850; in the second one, we considered only the impact of climatic change over time, while assuming no land use.

## 3. Results

**Figure 2:**
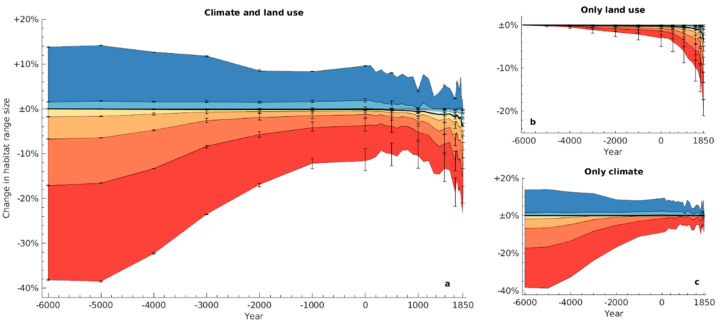
Estimated changes in range sizes between 6000 BCE and 1850 CE, relative to potential natural ranges in 1850. Coloured areas represent 10th, 20th, …, 90th percentiles (red, orange, …, blue) of range changes across the 16,919 species; the black line shows the across-species median. Panel (**a**) represents the default scenario accounting for both land use and climatic changes through time, whereas (**b**) is based on a constant global distribution of natural vegetation corresponding to climatic conditions in 1850, and (**c**) considers only the effects of climate through time, while assuming no anthropogenic land use. Upper and lower uncertainty bars in (**a**,**b**) correspond to the same analyses run based on the available upper and lower uncertainty bounds of the land use reconstructions.

Figure 2a shows the estimated changes in species’ range through time based on the derived reconstructions of past climate and land use. In the deeper past, the across-species median change in range size, relative to potential natural range sizes in 1850, was very small, ranging between 0% and −0.2% between 6000 BCE and 300 CE (Figure 2a, thick black line). However, whilst the median range did not change much, this was the result of a dynamic process in which many species did undergo range changes, as revealed by the increase in the variability of range size change as we go back in time. Towards the present, we observed a more consistent contraction of range sizes. By 1850, species had lost an estimated average of 3.6% of their potential natural ranges (Figure 2a, thick black line). This loss was coupled with an increase in the variability of species responses, as revealed by the broader variability of range size changes in Figure 2a.

Considering climate and land use effects separately provides insights into the drivers of these patterns. Changes in range sizes near the end of the study period were dominated by the impact of anthropogenic land use change, which increased approximately exponentially towards the Industrial era (Figure 2b; cf. also Figure 1a–c). The effects of climatic changes (Figure 2c), on the other hand, were not as consistently negative as those of land use. Our estimates suggest that climate-driven shifts in natural vegetation benefited some species while compromising others, similar to what has been observed in more recent times [5]. Despite the overall net effect being neutral across all species (the median is close to zero for the whole period of interest), the estimated impacts of pre-Industrial climate change on range sizes in recent millennia were considerable for some species. For 10% of species, climate change accounted for at least 37% smaller ranges at 6000 BCE compared to potential natural ranges in 1850 (Figure 2c, bottom edge of red patch), and for at least 12% larger ranges for another 10% of species (Figure 2c, top edge of dark blue patch). However, for most species, climate-driven changes were fairly small. Between 6000 BCE and 1850, the ranges of 52% of species varied within ±10% of the potential natural ranges in 1850 as the result of climatic changes (Figure 2c).

**Figure 3:**
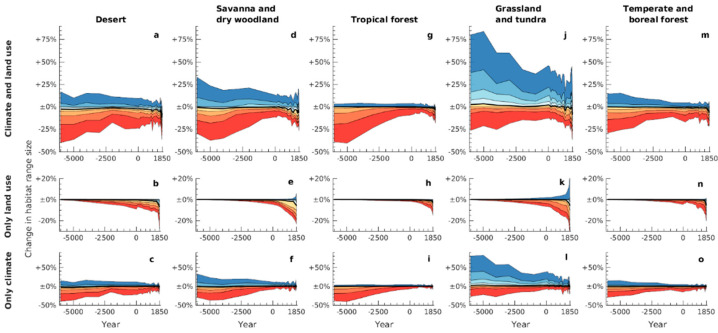
Estimated changes in range sizes over time, relative to potential natural ranges in 1850, depending on species’ primary megabiome. The top (**a**,**d**,**g**,**j**,**m**), middle (**b**,**e**,**h**,**k**,**n**), and bottom (**c**,**f**,**i**,**l**,**o**) panels in each column are analogous to Figure 2a–c, respectively, but represent only species with the relevant primary megabiome.

Our estimates suggest that range changes experienced by species were not homogenous across the world’s ecosystems. When considering species separately according to their primary megabiome (defined as one of the following categories that accounted for the largest area in a given species’ range in 1850 CE [5]: desert, savanna and dry woodland, tropical forest, grassland and tundra, or temperature and boreal forest), we found significant differences in how climatic change and land use affected species from different types of natural habitat (Figure 3). In particular, the ranges of a sizeable number of grassland species were substantially larger in the deeper past as the result of different climatic conditions, whilst ranges of many tropical species were smaller, compared to the present (Figure 3j,g). These results are consistent with our own simulations (Figure 1d) and earlier global palaeovegetation work demonstrating that pre-Industrial climatic change in recent millennia has increased the overall spatial extent of tropical forest and decreased the extent of grassland [26]. Other megabiomes experienced less pronounced changes [26], in alignment with our results for these habitats. Our analysis shows that species that benefited from anthropogenic land use change included a relatively large proportion of grassland species (Figure 3k), consistent with a relatively higher tolerance for cropland and pasture habitats in these species compared to others [27].

**Figure 4:**
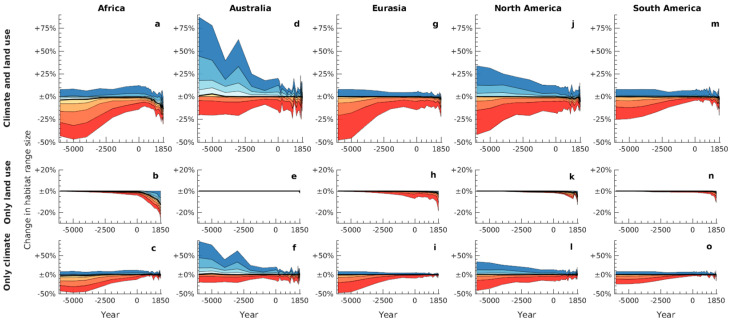
Estimated changes in range sizes over time, relative to potential natural ranges in 1850, depending on species’ primary geographical region. The top (**a**,**d**,**g**,**j**,**m**), middle (**b**,**e**,**h**,**k**,**n**), and bottom (**c**,**f**,**i**,**l**,**o**) panels in each column are analogous to Figure 2a–c, respectively, but represent only species with the relevant primary region.

Similarly, estimated range changes were not homogenous across global geographical areas. For five major regions (Africa, Australia, Eurasia, North and South America), we considered separately the range dynamics of species according to their primary geographical region, defined as the area that most of their potential natural range in 1850 CE overlapped with (Figure 4). We found, in particular, a relatively higher proportion of species whose ranges decreased in area towards the present in Australia, and somewhat less in North America, as the result of climatic changes (Figure 4d,f,j,l). Our analysis also highlight the effects of different land use histories in these regions on species’ ranges, specifically the comparatively later and less extensive conversion of natural habitat in Australia and South America, compared to other parts of the world (Figure 4b,e,h,k,n).

## 4. Discussion

During the period of interest (6000 BCE to 1850 CE), the distribution of the estimated species ranges was mostly at equilibrium, with no substantial change in the median. However, it is important to note that this equilibrium was dynamic, and a number of species changed their range significantly over the millennia. For most of the study period, this dynamism was mostly the result of climate change; even though the Holocene was relatively stable compared to the strong fluctuations that accompanied the last glaciation, the changes observed during the period of interest were sufficient to impact many species in a significant way. The impact of land use change on range sizes was limited for several thousand years following the advent of food production around 11,500 years ago. A sizeable effect on range sizes only occurred over the last two thousand years, when cropland and pasture area reached a significant magnitude.

A recent application of the same methodology used in this paper to the Industrial era [5] provids context for our results for pre-Industrial times. When compared to changes in the subsequent period up until the present, climate and land use-driven changes in species’ range sizes between 6000 BCE and 1850 CE were overall small for most species. To date, anthropogenic climate change and a rapid global intensification of land use since the beginning of the Industrial era are estimated to have caused an across-species median range loss of 18% and the loss of at least half of their potential natural range size for 16% of species [5]. This magnitude of estimated range contractions was far beyond what our data suggest occurred between 6000 BCE and 1850 CE. In particular, the fact that range changes induced by natural climatic variability over the past ~8000 years are dwarfed by the effects of current anthropogenic land use puts into perspective the tremendous negative impacts of human activity on the habitats of the world’s species during a relatively stable climatic period.

We refer to [5] for an in-depth discussion of the method used here to estimate species’ ranges through time, specifically of the assumptions that species are mobile enough to track long-term biome changes and that species’ past ranges vary within the available extents of occurrence, as well as of the role of the spatial resolution of the approach and that of uncertainties and biases in the available species occurrence and habitat data. In terms of caveats relevant specifically to the very long time frame considered here, we note that the available species data did not allow us to reconstruct whether, and if so when, there were changes in species’ habitat preferences over time. In particular, some species may have only gradually developed an ability to tolerate agricultural land cover. In such instances, true range losses due to land use change may have been higher in the deeper past than suggested by our analysis, before converging towards our estimates in more recent times. Whilst changes in the preferences of natural biome types over time are also possible in principle, these would have generally required major changes in species’ ecology, which, given the greater length of evolutionary time scales for mammals, birds, and amphibians in relation to the time scale considered here, we would not assume to be an important limitation of our analysis.

## 5. Conclusions

Here, we estimated the range size dynamics of 16,919 mammal, bird, and amphibian species from 6000 BCE until the beginning of the Industrial period, 1850 CE, based on changes in global climate and land use. Our results revealed that biome shifts driven by pre-Industrial climatic changes likely had important effects on species’ ranges, benefitting some whilst restraining others, whereas land conversion for human infrastructure and agriculture had a more consistently negative impact on species. Overall, changes in range sizes induced by these two factors were small compared to changes observed and projected for the recent past and imminent future, respectively, putting into context the magnitude of the impact that human activity has had on global biodiversity since the Industrial Revolution.

## Data Availability

Not applicable.

## Supplementary Materials

The following are available online at https://www.mdpi.com/article/10.3390/ani11123561/s1, Figure S1: Method of estimating potential natural and actual range for the example of the bat-eared fox (*Otocyon megalotis*) in the year 1850.
