# Supplementary material for: Range Sizes of the World’s Mammals, Birds, and Amphibians from the Mid-Holocene to the Industrial Period
Source: Animals (Basel). 2021 Dec 15;11(12):3561. doi: 10.3390/ani11123561 (PMC8698007; doi:10.3390/ani11123561)
Supplement: Supplementary file 1 [file animals-11-03561-s001.zip › animals-1472225-supplementary.pdf]

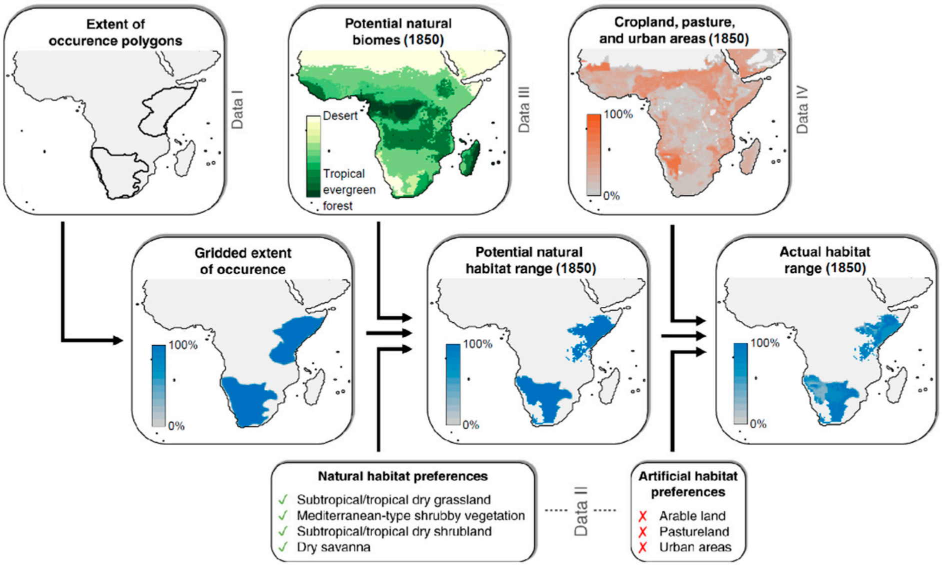

Figure S1. Method of estimating potential natural and actual range for the example of the bat-eared fox (*Otocyon megalotis*) in the year 1850. (Adapted from [5]). Extents of occurrence polygons are rasterised, then combined with potential natural biome reconstructions and natural habitat preferences to estimate potential natural habitat ranges, and finally combined with land use reconstructions and artificial habitat preferences to estimate actual habitat ranges. (Here, for visualisation purposes, cropland, pasture, and urban areas were aggregated into one map; in reality, our method checks each of them separately against species' artificial habitat preferences.)
